# Unlocking the Power: New Insights into the Anti-Aging Properties of Mushrooms

**DOI:** 10.3390/jof10030215

**Published:** 2024-03-14

**Authors:** Jing Luo, Kumar Ganesan, Baojun Xu

**Affiliations:** 1 Food Science and Technology Programme, Department of Life Sciences, BNU-HKBU United International College, Zhuhai 519087, China; jing.luo@tum-create.edu.sg (J.L.); kumarg@hku.hk (K.G.); 2 TUMCREATE, 1 Create Way, Singapore 138602, Singapore; 3 School of Chinese Medicine, Li Ka Shing Faculty of Medicine, The University of Hong Kong, Hong Kong, China

**Keywords:** Mushrooms, anti-aging, age-related disease, cellular mechanisms, bioactive compounds

## Abstract

Aging is a complex biological process that is influenced by both intrinsic and extrinsic factors. Recently, it has been discovered that reactive oxygen species can accelerate the aging process, leading to an increased incidence of age-related diseases that are characteristic of aging. This review aims to discuss the potential of mushrooms as a dietary intervention for anti-aging, focusing on their nutritional perspective. Mushrooms contain various bioactive compounds, including carbohydrates, bioactive proteins, fungal lipids, and phenolic compounds. These compounds have shown promising effectiveness in combating skin aging and age-related diseases. In vitro and in vivo studies have demonstrated that treatments with mushrooms or their extracts can significantly extend lifespan and improve health span. Furthermore, studies have aimed to elucidate the precise cellular and molecular mechanisms of action and the structure–activity relationship of mushroom bioactive compounds. These findings provide a strong basis for further research, including human clinical trials and nutritional investigations, to explore the potential benefits of mushrooms in real-life anti-aging practices. By exploring the anti-aging effects of mushrooms, this review aims to provide valuable insights that can contribute to the development of broader strategies for healthy aging.

## 1. Introduction

The global population is currently experiencing a significant expansion of aging populations compared to previous years. This trend is reflected in the increase in average life expectancy at birth, which has risen by 6.2 years from 65.3 years in 1990, to 71.5 years in 2013. Additionally, individuals who reach the age of 60 can now expect to live for another 22 years on average [1]. By the year 2040, it is projected that the average life expectancy will increase by 4.4 years for both men and women. Men can expect to live an average of 74.3 years, while women can expect to live an average of 79.7 years. However, these numbers may vary depending on individual health conditions [2]. As the population ages, there has been a noticeable increase in the prevalence of chronic degenerative diseases such as neurodegenerative and cardiovascular diseases, diabetes, and cancer. These diseases contribute to up to 70% of global mortality each year, including premature deaths occurring between the ages of 30 and 70 [1]. It is important to note that, while aging is often accompanied by deteriorative changes and an increased risk of functional declines or diseases, aging itself is not considered a disease. The focus of anti-aging strategies is not to reverse or halt the aging process, but rather to promote healthy aging and reduce the incidence of age-related diseases. The World Health Organization recommends adopting healthy dietary habits, engaging in regular physical activity, and controlling tobacco use as effective measures to alleviate or prevent the incidence of chronic diseases. By following these guidelines, the risk of developing age-related diseases can be reduced [3].

There is growing evidence to suggest that healthy aging can be promoted by consuming nutraceuticals and following various dietary patterns, such as caloric restriction, intermittent fasting, a Mediterranean diet, an Okinawan diet, and a Nordic diet. These dietary patterns have been evaluated for their negative correlation with aging and age-related conditions and diseases [4,5], which has led to a search for anti-aging components from food sources and an investigation of the underlying mechanisms of anti-aging pathways. Bioactive compounds derived from plant sources, including fruits and vegetables, roots, seeds, and edible flowers, have been suggested to exert anti-aging effects. These compounds include certain polysaccharides, phenolic compounds, and peptides [6,7]. In recent years, mushrooms—filamentous fungi with fruiting bodies—have also been shown to possess enormous pharmacological attributes that are valuable for healthy aging. These attributes include anti-oxidant, immunomodulatory, neuroprotective, anti-inflammatory, and anti-cancer properties [8,9,10,11].

Mushrooms are nutritious foods that are rich in carbohydrates and proteins, with a lower content of lipids [12]. In addition to their nutritional value, mushrooms contain various bioactive compounds, such as β-glucans, lectins, and linolenic acids, which can be isolated through different extraction methods. These compounds confer a variety of pharmacological activities and may enhance the immune system and strengthen the biological function of the body [13]. Regular intake of mushrooms or their extracts may help alleviate age-related diseases. This review focuses on the anti-aging properties of mushrooms from the perspective of aging and age-related diseases, with a brief introduction of the major bioactive compounds found in edible and medicinal mushrooms.

## 2. Aging

### 2.1. Aging and Age-Related Diseases

Aging is a complex process that involves the time-dependent accumulation of diverse deleterious changes in cells, tissues, organs, or systems that increase vulnerability to chronic illness and death [14,15]. Nine candidate hallmarks of aging have been identified and classified, including primary hallmarks (genomic instability, telomere attrition, epigenetic alterations, and loss of proteostasis), antagonistic hallmarks (deregulated nutrient sensing, mitochondrial dysfunction, and cellular senescence), and integrative hallmarks (stem cell exhaustion and altered intercellular communication), all of which are correlated with each other [16]. The antagonistic hallmarks exert positive effects at low levels but negatively affect the organism at high levels [16]. For example, reactive oxygen species (ROS) are important signaling molecules that play a role in regulating cellular functions, but excessive levels can lead to oxidative damage and contribute to aging. The primary hallmarks are the contributors to molecular damage during aging, while the integrative hallmarks are signs of failure of cellular homeostasis and metabolism mechanisms to ameliorate the damage. These hallmarks are interconnected with each other and could serve as a guidance to decipher the mechanistic molecular basis for prolonging health span and development of strategies for longevity, such as stem-cell-based therapies, epigenetic drugs, anti-inflammatory drugs, and dietary restrictions [16].

The free radical theory of aging, proposed in 1956 by Denham Harman [17], is a widely accepted theory of aging. The theory postulates that the aging process is triggered by the initiation of free radical reactions, leading to increased generation of free radicals by damaged mitochondria with increasing age [18]. Major sources of free radical reactions in mammals include non-enzymatic reaction of oxygen, ionizing radiation, cytochrome P-450 system, respiratory chain, phagocytosis, and prostaglandin synthesis, which lead to the accumulation of oxidative damage and may shorten the lifespan. Several defenses that alleviate the damage of the reactions include DNA repair mechanisms, superoxide dismutase, glutathione peroxidase, and anti-oxidants (e.g., carotenes and vitamin E) [15,19].

ROS are byproducts of oxidative metabolism that can induce cellular defense mechanisms against oxidative invasion at low doses, potentially prolonging health span and lifespan. However, long-term excessive exposure to ROS can lead to the oxidation of nucleic acids, proteins, and lipids, causing damage to macromolecules and mitochondrial dysfunction. This can disrupt cell homeostasis and result in cellular death [20]. ROS production is driven by progressive mitochondrial dysfunction with increasing age, creating a positive feedback loop of ROS generation and oxidative damage accumulation [18]. Concurrently, oxidative stress arises due to excessive ROS levels and limited anti-oxidant defense capability, leading to cellular senescence and a shortened lifespan. The accumulation of oxidative damage to macromolecules and mitochondria contributes to detrimental consequences, such as pathophysiological changes, functional decline, and accelerated aging, which are associated with age-related conditions such as inflammation, cardiovascular diseases, neurodegenerative diseases, autoimmune diseases, and cancer [21].

**Figure 1:**
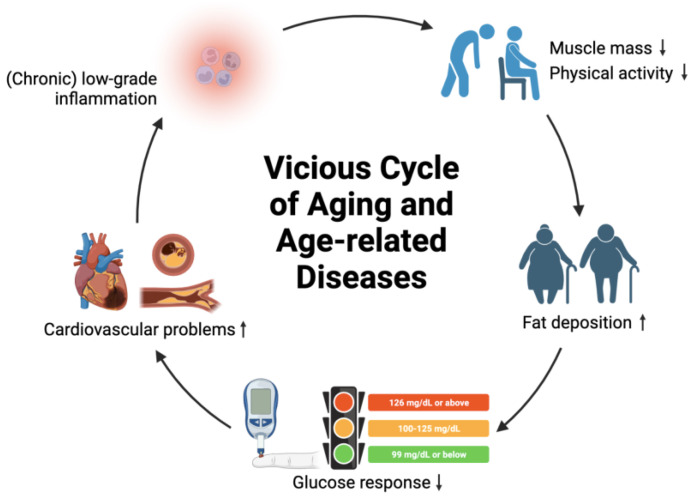
Concept of vicious cycle of aging and age-related diseases. Symbol ↑ denotes increase; symbol ↓ denotes decrease.

It is important to note that aging itself is not a disease. Age-related diseases can be considered “symptoms” of aging, initiated by minor disturbances that are intensified via vicious positive feedback loops, destabilizing the physiology of an organism and potentially leading to destruction (i.e., mortality) if no negative feedback loops are in place [22]. For example, low-grade inflammation can intensify in chronic inflammation, leading to decreased muscle mass, decreased physical activity, and excess fat deposition. This can further contribute to obesity, diabetes, and cardiovascular problems. Eventually, cardiovascular diseases can arise and worsen the physiological status of an individual by triggering chronic inflammation. To minimize cumulative damage to different organs and maintain cell function for healthy aging, interventions that can interrupt or break the vicious cycles of age-related diseases can be implemented, including medications, lifestyle adjustments, and dietary management (Figure 1).

### 2.2. Aging and Dietary Intervention

The lifestyle of an individual is closely linked to their health span and lifespan. One of the main ways to modify lifestyle for better health maintenance and to reduce the incidence of age-related diseases is through dietary management. Unhealthy dietary habits and lifestyle can accelerate the aging process by causing molecular and cellular damage. For example, a sedentary lifestyle, combined with a “Western diet”, that is high in energy but lacking in nutrition, has been associated with reduced lifespan and increased occurrence of age-related conditions such as obesity, type 2 diabetes, and cancer [23]. On the other hand, caloric restriction (CR) has been shown to slow down the rate of aging and extend health span. CR involves reducing total energy intake by 20% to 40% while ensuring optimal nutrition, compared to an ad libitum diet. This approach has been demonstrated to extend lifespan and health span in various experimental models, including yeast, fruit flies, mice, nonhuman primates, and even humans [24,25,26].

According to the theory of aging, CR enhances longevity by reducing oxidative damage and increasing resistance to oxidative stress through specific signaling pathways. The stress caused by CR, such as nutrient deprivation, activates defense mechanisms against oxidative damage, thereby slowing down the aging process [27]. CR also affects physiological pathways that may mediate anti-aging effects, such as the insulin-like growth factor-1 and insulin signaling pathways, the mammalian target of rapamycin (mTOR) pathway, and the sirtuins pathway [24,28,29]. Previous studies have demonstrated the potential of implementing CR as an anti-aging regimen, as adherence to this dietary management reduces biomarkers associated with the development of age-related diseases, including cardiovascular diseases, autoimmune disorders, neurodegenerative diseases, diabetes, and cancer [29,30,31]. Therefore, CR can be considered as the mechanistic foundation for healthy aging strategies involving dietary intervention, which can prolong lifespan and maintain physiological function for an extended health span.

Despite the potential benefits of CR, it can be challenging for individuals to adhere to it in the long term due to various pitfalls and health concerns, such as hypotension, osteoporosis, slower wound healing, depression, and irritability [32]. As a result, scientists have explored alternative diet regimens and studied different dietary patterns that may offer similar benefits to CR but are more feasible for humans to sustain. One such approach is intermittent fasting, which shares the same concept as CR. Intermittent fasting activates cellular pathways that enhance the body’s intrinsic defense against oxidative stress, promotes the removal of damaged molecules, and facilitates tissue repair and growth. It also helps to suppress inflammation and improve stress resistance [33,34].

In addition to dietary modifications, researchers have developed anti-aging drugs that mimic the effects of CR. Examples include rapamycin and metformin, which have shown promising effects in various model organisms and clinical trials. Rapamycin delays aging by inhibiting mTOR, thereby maintaining the normal functioning of mitochondria and stem cells. Metformin, on the other hand, affects telomere length, reduces oxidative damage to DNA, and modulates the synthesis and degradation of age-related proteins [35,36]. However, it is important to note that there are concerns and side effects associated with the use of these drugs. For instance, rapamycin may lead to nephrotoxicity and thrombocytopenia, while metformin may cause vitamin B12 deficiency and lactic acid accumulation [37,38]. Therefore, there is a need to explore naturally occurring compounds that have significant anti-aging effects with minimal side effects.

Nutraceuticals and dietary supplements are also viable alternatives for anti-aging and extending health span. Examples include curcumin, quercetin, ginseng, and medicinal mushrooms, which exhibit anti-inflammatory, immunomodulatory, and antioxidative effects [39,40,41]. A diet rich in fruits and vegetables, which provide a significant number of nutraceuticals and phytochemicals, is crucial for maintaining overall health. Interestingly, mushrooms, although not classified as animals or plants but as part of the fungal kingdom, are often considered as vegetables. They are low in calories, sodium, and fat, while being a valuable source of fiber, phenolic compounds, β-glucans, selenium, glutathione, B vitamins, and vitamin D. These components serve as protective agents against oxidative damage, which accelerates aging [12]. Medicinal mushrooms have also been used for centuries in traditional therapies, like Chinese medicine and Indian Ayurveda medicine, to alleviate symptoms of various diseases [42]. The bioactive compounds found in mushrooms may contribute to their anti-aging effects through various physiological pathways involved in aging and age-related diseases.

### 2.3. Ageing, Mental Health and Gender

Gender and mental health can significantly impact ageing experiences. Gender influences ageing in various ways, including health outcomes, social roles and expectations, and economic status. Women are more likely to experience depression, anxiety, and stress due to factors such as caregiving responsibilities, hormonal changes, and discrimination [43]. Women also tend to report higher levels of loneliness and social isolation in later life. In contrast, men may experience social isolation and mental health issues due to societal expectations of masculinity, which can lead to reluctance in seeking help for mental health problems [44].

Gender differences in health outcomes are well-documented, with women living longer but experiencing more chronic health conditions than men. Women are more likely to experience osteoporosis, urinary incontinence, and depression than men. Women also experience menopause, which can lead to physical and psychological symptoms [45]. Men, on the other hand, are more likely to experience heart disease, stroke, and certain types of cancer. Biological factors such as sex hormones, genetics, and lifestyle factors like diet, exercise, and smoking influence gender differences in health outcomes [46].

Gender roles and expectations can influence ageing experiences [47]. Women are often expected to take on caregiving roles for children, spouses, or ageing parents, which can lead to stress and impact their own health and well-being. Women may also face ageism and discrimination in the workplace, leading to financial insecurity in later life. Men, on the other hand, may experience pressure to maintain their independence and financial stability, leading to social isolation and mental health issues [43,44,48].

Gender differences in economic status can also impact ageing experiences. Women often earn less than men over their lifetimes, leading to lower retirement savings and financial insecurity in later life. Women are also more likely to work part-time or take career breaks to care for children or ageing parents, which can impact their pension entitlements. This can lead to poverty and social exclusion in later life [47,49]. Mental health issues, such as depression, anxiety, and cognitive impairment, can also impact ageing experiences. Depression is a common mental health issue among older adults and can lead to social isolation, physical illness, and suicide. Anxiety can affect quality of life and daily functioning. Cognitive impairment, including dementia, can result in memory loss, decision-making difficulties, and loss of independence and increased caregiving needs [50].

Studies have shown that gender and mental health can interact to influence ageing experiences [28,36,43,44,51,52]. Women with depression may be more prone to physical disability and cognitive decline in later life compared to men with depression. Similarly, men with higher levels of anxiety may be more likely to experience cognitive decline than women with anxiety [43]. Addressing gender and mental health in ageing policies and practices is crucial to ensure that older adults receive appropriate support and services. This includes promoting gender equity, addressing mental health stigma, and providing accessible and affordable mental health care for older adults [22].

## 3. Components of Mushrooms and Their Anti-Aging Effects

Mushrooms have long been recognized for their nutritional value and potential health benefits. Edible mushrooms are not only rich in protein, fiber, vitamins, and minerals but also have low levels of fat, making them highly nutritious [53,54]. They contain all the essential amino acids and have a higher protein content compared to most vegetables, making them particularly beneficial for vegetarians. In addition to their nutritional value, edible mushrooms, as fungi, have the ability to produce a wide range of chemical compounds known as mycochemicals. These mycochemicals can act as bioactive substances with various advantages for human health [55]. Mushrooms have been found to contain significant levels of mycochemicals that serve as bioactive compounds, offering a range of health benefits against aging and age-related diseases [53,54].

### 3.1. Bioactive Compounds in Mushrooms

Bioactive compounds extracted from mushrooms have been extensively studied for their ability to enhance cellular functions and provide health benefits. The following text summarizes four representative categories of bioactive compounds found in mushrooms: carbohydrates, proteins, lipids, and phenolic compounds.

#### 3.1.1. Carbohydrates

Carbohydrates derived from mushrooms have been extensively studied for their anti-tumor, anti-inflammatory, and immunomodulatory activities [56,57]. Numerous monosaccharides found in mushrooms, including arabinose, fructose, fucose, galactose, glucose, mannose, mannitol, rhamnose, trehalose, and xylose, have been identified as exhibiting these activities. They primarily achieve this through the activation of cytokines, such as interferons and interleukins, and involve cellular pathways that include dendritic cells, natural killer cells, neutrophils, and cytotoxic macrophages [57,58]. β-Glucans, the main type of carbohydrates found in mushrooms, have been shown to possess antioxidative, anti-cancer, immunomodulatory, and neuroprotective properties. They are considered potent agents for stimulating the immune system and protecting against carcinogens, pathogens, and toxins [59,60,61,62,63,64]. The biological activity and health benefits of β-glucans isolated from mushrooms, particularly in relation to immune health, are crucial for healthy aging. Supplementation with mushroom carbohydrates, which contain β-glucans, could be an effective strategy for anti-aging. Table 1 provides a list of various mushrooms that contain bioactive carbohydrates.

#### 3.1.2. Proteins

Compared to other food sources, mushrooms contain higher levels of bioactive proteins such as lectins, ribosome inactivating proteins, fungal immunomodulatory proteins, and laccases, which possess various biological activities (Table 2) including antioxidative, immunomodulatory, anti-inflammatory, and anti-cancer properties [90]. Lectins are non-immune proteins or glycoproteins that bind to specific carbohydrates on cell surfaces, acting as nutraceuticals with immunomodulatory, anti-tumor, and anti-proliferative properties [90]. Other mushroom proteins, such as laccase, fungal immunomodulatory protein, and ribosome inactivating proteins, have distinct bioactive activities. Laccases are considered multicopper oxidases implicated in processes such as pathogenesis, morphogenesis, and immunogenesis of an organism [90]. Fungal immunomodulatory proteins purified from mushrooms, such as *Ganoderma lucidum*, *Ganoderma tsugae*, *Poria cocos*, *and Trametes versicolor*, have been suggested as potential adjuvants for tumor therapy due to their structural similarity to human antibodies and their ability to suppress tumor metastasis and invasion [91,92,93,94,95].

The ribosome inactivating protein family acts as rRNA N-glycosylase, inactivating 60S ribosomal subunits through an N-glycosidic cleavage that eliminates one or more adenosine residues from rRNA to inhibit protein synthesis [112]. Members of the ribosome inactivating protein family, such as trichosanthin, luffin, ricin, and abrin, have been of considerable interest due to their potent activity against viral infections and their potential use as immunotoxins for cancer treatment by conjugating with monoclonal antibodies [113,114,115]. However, it is noteworthy that some mushroom ribosome inactivating proteins may be hazardous and pose adverse effects on health. For instance, hypsin from *Hypsizigus mamoreus* has been reported to increase in vitro cell death [116]. Therefore, it is important to elucidate the structure-functional properties of mushroom proteins as they may be toxic to humans when consumed. Table 2 lists various bioactive proteins derived from mushrooms.

#### 3.1.3. Lipids

Although mushrooms have a low fat content ranging from 0.1% to 16.3%, they are a good source of high-quality essential fatty acids such as oleic acid (1–60.3% of total fatty acids in 100 g), linoleic acid (0–81.1% of total fatty acids in 100 g), and linolenic acid (0–28.8% of total fatty acids in 100 g) [117]. Table 3 summarizes the lipid profiles of various mushrooms in terms of the content of saturated fatty acids (SFA), monounsaturated fatty acids (MUFA), and polyunsaturated fatty acids (PUFA). Mushrooms are good sources of unsaturated fatty acids, as observed in a study by Günç Ergönül et al. [118] who investigated the fatty acid compositions of six wild edible mushroom species and found that unsaturated fatty acids predominated over saturated ones. In most nutritional characterization studies, mushroom fatty acids are commonly determined using gas-liquid chromatography coupled with a flame ionization detector. However, the sample extraction method used prior to measurement may impact the final outcome of lipid profiles. For instance, a study by Sinanoglou et al. [119] investigated the lipid profiles of *Laetiporus sulphureus* using different combinations of extraction methods and two individual solvents and found variations among the four combinations [119]. Ergosterol, the major sterol found in mushrooms, accounts for the major lipid component of fungal extracellular vesicles as well [120]. Ergosterol extracted from medicinal mushroom *Ganoderma lucidum* has been shown to exert anti-oxidant effects and reduce the risk of cardiovascular diseases while extending lifespan [55,121,122]. Compared to lipids from animal sources, edible mushrooms are advantageous due to their high levels of polyunsaturated fatty acids, which may regulate various physiological functions in age-related diseases, such as decreasing blood pressure and triglyceride levels, and reducing the risks of age-related cardiovascular diseases, arthritis, and neurodegenerative diseases [64,123]. Therefore, mushrooms may play a significant role in human nutrition and anti-aging regimens based on their fatty acid profiles.

#### 3.1.4. Phenolic Compounds

Phenolic compounds found in mushrooms are typically considered secondary metabolites. The most prominent phenolic compounds in mushrooms include heteroglycans, lectins, phenolic acids (such as ferulic, gallic, and cinnamic acids), flavonoids (including hesperetin, quercetin, kaempferol, and naringenin), steroids, alkaloids, tannins, chitinous substances, terpenoids, and tocopherols. These compounds exhibit various biological activities, including anti-oxidant, anti-tumor, anti-inflammatory, anti-hyperglycemic, anti-osteoporotic, anti-tyrosinase, and anti-microbial effects, primarily due to their strong antioxidative properties [125,126,127,128]. Some of the preferred mushroom species for extracting phenolic compounds include *Agaricus brasiliensis* (almond mushroom), *Cantharellus cibarius* (chanterelle), *Lactarius indigo* (indigo milk cap), *Inonotus obliquus* (chaga mushroom), and *Melanoleuca cognate* [126,129,130,131]. Table 4 provides a summary of representative phenolic compounds extracted from various mushroom species.

## 4. Effects of Mushrooms and Their Anti-Aging Properties

Indeed, numerous studies have investigated the composition of mushrooms and their potential anti-aging effects. Various components extracted from mushrooms, including polysaccharides, phenolics, terpenes, lipids, vitamins, and minerals, have been found to possess anti-oxidant, anti-wrinkle, and anti-aging properties [152,153]. However, it is important to note that the anti-aging effects of mushrooms are primarily focused on skin aging and age-related diseases. The following provides an overview of these two aspects. The disruption of the collagen and elastin network in the skin due to excessive oxidative stress or free radicals is a characteristic of aging. As a result, anti-aging cosmetics are developed to repair and maintain the skin barrier. Many studies have highlighted the potential of bioactive compounds derived from mushrooms to serve as anti-aging ingredients in serums, topical creams, and other cosmetics, primarily due to their anti-oxidant and anti-wrinkle properties [10,58,154,155,156,157,158,159,160,161,162]. These compounds can help protect the skin from oxidative damage, reduce the appearance of wrinkles, and improve overall skin health.

### 4.1. Anti-Oxidant Activity

Oxidative stress is a condition that occurs when there is an imbalance between the production of ROS and the body’s ability to neutralize them with anti-oxidants. ROS can damage cellular components, including DNA, proteins, and lipids, leading to cellular dysfunction and aging. Mushrooms have been investigated for their potential anti-oxidative properties and their ability to mitigate oxidative stress [163,164]. The anti-oxidant activity of mycochemicals derived from mushrooms plays a significant role in the defense and repair systems against oxidative damage and free radicals, which accelerate the aging process. Extracts from shiitake mushrooms (*Lentinula edodes*) have been found to act as inducers of anti-oxidant enzymes, such as glutathione peroxidase and superoxide dismutase. These enzymes stimulate the conversion of myofibroblasts to fibroblasts, reversing fibrosis and protecting the skin from oxidative damage [154]. Furthermore, L-ergothioneine, isolated from shiitake mushrooms, has been shown to scavenge free radicals, particularly those affecting the mitochondrial membrane, thus reducing oxidative stress on the skin [155]. L-ergothioneine, a thiourea derivative of histidine, is found in high concentrations in various mushrooms, including *Pleurotus ostreatus* (oyster mushroom), *Pleurotus eryngii* (King oyster mushroom), brown *Agaricus bisporus* (brown button mushroom), and *Grifola frondose* [155]. Additionally, mushroom glucan, extracted from *Phellinus ribis* and the somatic hybrid mushroom of *Pleurotus florida* and *Calocybe indica* var. APK2, has been found to activate immune cells and act as an anti-aging and anti-oxidant agent for the skin [156,157]. The anti-oxidant properties of mushrooms have also been demonstrated by *Ganoderma lucidum* (lingzhi) and *Phellinus linteus* (black hoof mushroom) in both in vitro assays and in vivo when consumed as food [58,158].

Mushrooms are rich in various anti-oxidants, including phenolic compounds, polysaccharides, and ergothioneine, that can scavenge free radicals and reduce oxidative damage. For example, polysaccharides from mushrooms such as *Grifola frondosa* (maitake), *Agaricus blazei* (almond mushroom), and *Pleurotus ostreatus* (oyster mushroom) have been shown to possess potent anti-oxidant activity [155,165,166]. A study retrospectively examined 37 participants who underwent a dietary intervention featuring daily consumption of 100 g of *A. bisorus* for 16 weeks [165]. Significant improvements in serum markers associated with inflammation and oxidative stress were observed after 16 weeks, including increases in ergothioneine levels and oxygen radical absorption capacity and reductions in oxidative stress-inducing factors carboxymethyllysine and methylglyoxal, suggesting potential anti-inflammatory and anti-oxidant benefits of *A. bisporus* consumption [165]. Ethanolic extract of oyster mushroom demonstrated potent radical-scavenging activity. At a maximum concentration of 10 mg/mL, the extract showed the highest level of radical-scavenging activity, with scavenging rates of 56.20% and 60.02% observed for hydroxyl and superoxide radicals, respectively [151]. The results show great potential of oyster mushroom as a readily available source of natural anti-oxidants for dietary supplementation or pharmaceutical use. Ergothioneine, also found in various mushrooms like *Pleurotus eryngii* (king trumpet mushroom) and *Lactarius deliciosus* (saffron milk cap), has been shown to have powerful anti-oxidant properties and reduce oxidative stress [84,87,151,167].

Mushrooms have also been found to protect against oxidative stress-induced damage to mitochondria, the organelles responsible for energy production within cells. For instance, polysaccharides from *Cordyceps sinensis* (caterpillar fungus) have been shown to enhance mitochondrial function and reduce oxidative damage in aging mice [39,168,169]. This suggests that mushroom bioactive compounds may help preserve mitochondrial function and mitigate age-related decline. Mushroom bioactive compounds can modulate signaling pathways involved in oxidative stress. For example, polysaccharides from mushrooms such as *Ganoderma lucidum* (lingzhi) and *Lentinula edodes* (shiitake) have been found to inhibit the production of ROS and increase the activity of anti-oxidant enzymes, such as SOD and CAT [77,158,170,171]. By regulating ROS production and anti-oxidant enzyme activity, mushrooms may help reduce oxidative stress and associated tissue damage.

### 4.2. Anti-Wrinkle Effects

One of the primary signs of skin aging is the formation of wrinkles, which is primarily caused by the loss of structural proteins in the dermis and elastase-induced degradation of elastin, leading to the expression of matrix metalloproteinases [159]. Lee, Lee, Kim, Yoo and Yang [10] discovered that Clitocybin A, an isoindolinone derived from the Korean mushroom *Clitocybe aurantiaca*, exhibited scavenging activity against ROS and inhibitory effects on elastase in human primary dermal fibroblast-neonatal cells. This suggests the potential of clitocybin A as an effective ingredient in anti-wrinkle cosmetic products. Similarly, the extract of the mycelium of the pine mushroom (*Tricholoma matsutake*) was found to inhibit elastase activity and the expression of matrix metalloproteinases in human fibroblasts [160]. In addition to the factors mentioned above, targeting the pro-inflammatory enzyme cyclooxygenase-2 (COX-2) may also be a strategy for anti-wrinkle treatments. COX-2 is associated with the production of ROS and inflammation in normal skin tissue. Therefore, COX-2 inhibitors are applied in anti-wrinkle cosmetics [161].

Notably, several bioactive compounds extracted from mushrooms have been found to effectively inhibit COX-2 activity. Stanikunaite, Khan, Trappe and Ross [161] reported that the ethanol extract of fruiting bodies of the truffle-like fungus *Elaphomyces granulatus* exhibited a 68% inhibition of COX-2 activity at a concentration of 50 mg/mL in mouse macrophages (RWA 264.7). Further investigation led to the identification of two bioactive compounds in *E. granulatus*, namely syringic acid and syringaldehyde acid, which were suggested to be responsible for the COX-2 inhibitory property [161]. Moreover, an extract of *Ganoderma lucidum* containing spores and fruiting bodies in a ratio of 30:8 was found to attenuate UV-induced epidermis thickening and inhibit the expression of COX-2 in non-tumor skin tissues of mice. This highlights the potential of the extract as a key component in cosmetic products for skin maintenance [162].

### 4.3. Immunomodulatory Effects

Immunosenescence refers to the gradual deterioration of various components in the immune system due to natural age advancement, which can lead to irregular immune responses against viruses or pathogens and increased vulnerability to illnesses such as chronic inflammation, autoimmune diseases, and cancer [172]. Reinforcing the immune system is essential for longevity. Extracts of the medicinal mushroom *Agaricus blazei* Murill have been found to enhance the functions of phagocytic cells [8], contributing to anti-tumor effects by strengthening innate immunity. When exposed to *A. blazei* Murill extracts, the phagocytic cells interact and remove invasive pathogens, further triggering innate and adaptive immune responses through the release of chemokines and cytokines. Short-term oral supplementation of the extracts at doses of 0.5–5% has been shown to exert an immunostimulatory effect characterized by increased secretion of cytokines in whole blood [173]. 1,3-β-Glucans found in medicinal mushrooms are effective in stimulating the immune system by modulating T cells, macrophages, and natural killer cells, along with the production of cytokines [174].

Several edible mushrooms, including *Agaricus bisporus*, *Flammulina velutipes*, *Lentinus edodes*, *Pleurotus florida*, and *Trametes pubescens*, have been found to possess anti-inflammatory properties, as assessed by levels of lipopolysaccharide and interferon that activate macrophages, indicating their immunomodulatory ability [9,175,176]. Additionally, the secondary metabolite lectin purified from *Latiporus sulphureus* could promote immune cell proliferation and phagocytosis and activate cytokines, suggesting its potential immunopotentiation in pharmacology and functional foods [177]. The use of cultured Sanghuang mushroom (*Inonotus sanghuang*) extracts at doses of 8 mg/kg or 16 mg/kg in immunodeficient mice has exhibited immunoregenerative functions, suggesting the potential of these extracts as an alternative for nutraceutical medicine concerning cancer chemotherapy [178]. From a more recent point of view, the bioactivity of mushrooms is closely related to its interaction with the gut microbiota, where gut microbial metabolites play a key role in bridging the gap between immunomodulatory effect of mushrooms and the host after consumption. Vlassopoulou et al. [179] selected *Pleurotus eryngii* as a substrate for in vitro fermentation using gut microbiota sampled from healthy elderly volunteers. The fermentation supernatants, which comprised a group of gut microbial metabolites, were subjected to cellular assays in U937-derived human macrophages. Interestingly, improved immune response was observed in treatment of gut microbial fermentation supernatants from each individual, characterized by altered gene expression and levels of pro- and anti-inflammatory cytokines in the macrophages, and further verified using peripheral blood mononuclear cells of the volunteers [179]. Boulaka et al. [180] also assessed the immunomodulatory property of *P. eryngii* through in vitro fermentation using fecal sample collected from both male and female elderly subjects. While not observed in pre-fermentation supernatant treatment, post-fermentation supernatant exhibited protective effects against mitomycin C-induced DNA damage for human lymphocytes in a dose-dependent manner, suggesting its significant role in maintaining genome integrity via metabolites-gut microbiome-host interaction during aging, which attributes to immunomodulatory and anti-oxidant activities [180].

### 4.4. Cardioprotective Effects

**Figure 2:**
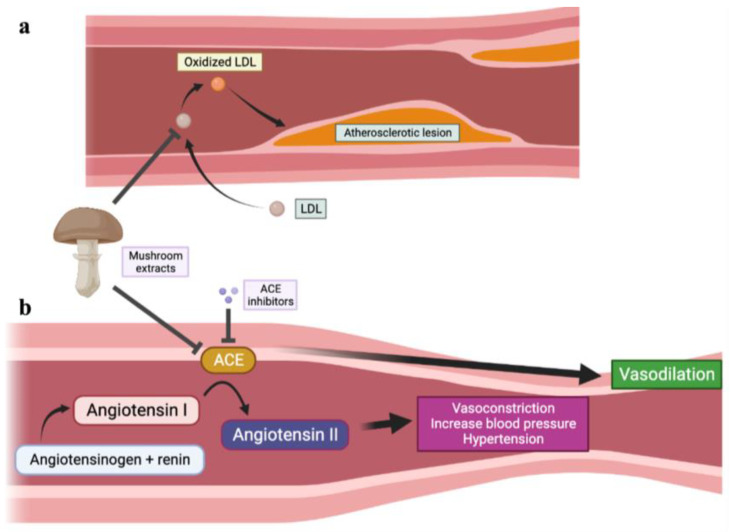
Schematic illustration of effects of mushroom extracts against cardiovascular diseases regarding lowering blood LDL and blood pressure. (**a**) Application of mushroom extracts decrease LDL levels and inhibit platelet aggregation. (**b**) Application of mushroom extracts inhibit ACE activity similarly to ACE inhibitors, thus leading to vasodilation and lower blood pressure. ACE, angiotensin converting enzyme. LDL, low-density lipoprotein.

The circulatory system is essential for the transportation of oxygenated blood and nutrients to tissues and organs. The aging process can significantly impact the cardiovascular system, leading to the development of cardiovascular diseases such as hypertension, cardiac hypertrophy, atherosclerosis, myocardial infarction, and stroke [181]. One of the factors responsible for high blood pressure and cardiac hypertrophy is the vasopressor octapeptide angiotensin II (Figure 2b), which is converted from angiotensin I in the presence of angiotensin I converting enzyme [167]. D-glucopyranose mannitol extracted from the mushroom *Pleurotus cornucopiae* (Tamogi-take mushroom) has been found to alleviate hypertension in spontaneously hypertensive rat models by inhibiting angiotensin I converting enzyme and lowering blood pressure [167,182]. Similarly, bioactive peptides extracted from the fruiting body of *Tricholoma matsutakei* also disrupt the function of angiotensin responsible for hypertension [183]. Atherosclerosis, a disease commonly associated with hypercholesterolemia, high levels of low-density lipoprotein (LDL) (Figure 2a), and low levels of high-density lipoprotein (HDL), is prevalent among older populations and poses risks of stroke. Regularly consuming mushrooms has been shown in various animal studies to have significant benefits in reducing hypertension, atherosclerosis, dyslipidemia, inflammation, and obesity [184,185].

Several mushrooms with medicinal properties, including *Hypsizygus marmoreus* (bunashimeji), *Grifola frondosa* (maitake), and *Pleurotus eryngii* (eringi), show potential in treating atherosclerosis [186,187]. In an atherosclerosis mouse model, the application of mushroom extracts decreased the incidence of atherosclerosis lesions, suggesting their potential use in treatment [187]. In a rat model fed a high-cholesterol diet, oral administration of *Pleurotus florida* powder extracts increased fecal lipid excretion while effectively decreasing serum triglycerides, total cholesterol, LDL, and very low-density lipoprotein levels when compared to control mice [186]. Additionally, the ethanol extract of lion’s mane mushroom and hot water extract from the mycelia of *Cordyceps sinensis* (caterpillar fungus) can enhance lipid metabolism by suppressing platelet aggregation, lowering LDL levels, and increasing HDL levels, acting as therapeutic agents for atherosclerosis and potentially decreasing the risk of myocardial infarction [61,188].

### 4.5. Neuroprotective Effects

Brain aging is a significant risk factor for neurodegenerative diseases and cognitive decline, including dementia, Alzheimer’s disease (AD), and Parkinson’s disease (PD). Excessive oxidative stress is a major contributor to brain aging. Research has been conducted on the effects of mushroom extracts on the oxidative state of the brain during aging. For example, an aqueous extract of *Agaricus blazei* was found to maintain the ROS levels in the brain of rats at a level that did not accelerate brain aging when administered daily at a dose of 50 mg/kg [189]. However, long-term and continuous treatment with the extract showed a tendency to be less effective in rats aged above 12 months, suggesting that intermittent treatment with short-term doses may be more beneficial [189]. In experiments using the roundworm *Caenorhabditis elegans*, an ethanolic extract of cloud ear fungus (*Auricularia polytricha*) attenuated glutamate-induced cytotoxicity and increased the expression of anti-oxidant enzyme genes, promoting longevity and health in the worms [190]. This suggests that cloud ear fungus could serve as a natural source of neuroprotective and anti-brain-aging agents.

**Figure 3:**
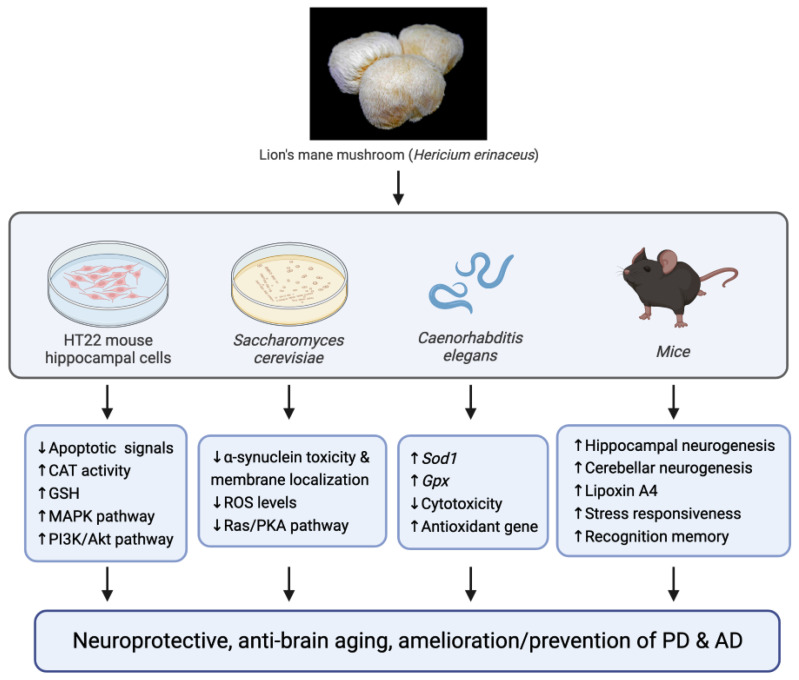
Anti-brain aging properties of Lion’s mane mushroom (*Hericium erinaceus*) observed in four experimental models. CAT, catalase; GSH, glutathione; MAPK, mitogen-activated protein kinase; PI3K, phosphatidylinositol 3-kinase; Akt, protein kinase B; ROS, reactive oxygen species; Ras/PKA, Ras protein/protein kinase A; *Sod1*, Cu/Zn superoxide dismutase; *Gpx*, glutathione peroxidase. Symbol ↑ denotes increase; symbol ↓ denotes decrease.

Figure 3 illustrates the neuroprotective properties of lion’s mane mushroom in four preclinical study models. Ethanol extracts of *H. erinaceus* demonstrated neuroprotective effects in mouse hippocampal neurons and microglia, protecting against oxidative damage and inflammation [191]. In the context of PD, *H. erinaceus* and *Grifola frondosa* (maitake mushroom) extracts have shown anti-aging effects in yeast by reducing α-synuclein toxicity and levels of ROS, as well as lowering α-synuclein membrane localization [192]. *H. erinaceus* has also shown beneficial effects in improving cognitive function and behavioral deficits in animal models of AD, as well as enhancing recognition memory and inducing neurogenesis in frail aging mice [193,194]. While studies have indicated the neuroprotective effects of edible and medicinal mushrooms, it is important to carefully verify their efficacy and potential adverse effects in human trials as the effects may vary.

### 4.6. Anti-Diabetic Effects

According to recent research, age-related type 2 diabetes is primarily caused by pathological changes in pancreatic beta cells. These changes include decreased proliferation and regeneration potential, disrupted transcriptome and proteostasis, increased accumulation of senescent cells, and the impact of systemic environmental stress. These factors result in the loss of functional cell mass and impaired insulin secretion and action [195]. Mushrooms, specifically polysaccharides like β-glucans, have been found to play a role in restoring pancreatic function. They boost insulin secretion by pancreatic beta cells, lower blood glucose levels, and improve the insulin response in peripheral tissues [196]. Exopolysaccharides isolated from cultured mycelium of *Phellinus baumii* and *Tremella fuciformis* (snow fungus) have shown blood glucose-lowering effects in mice with obesity-induced diabetes [197].

**Figure 4:**
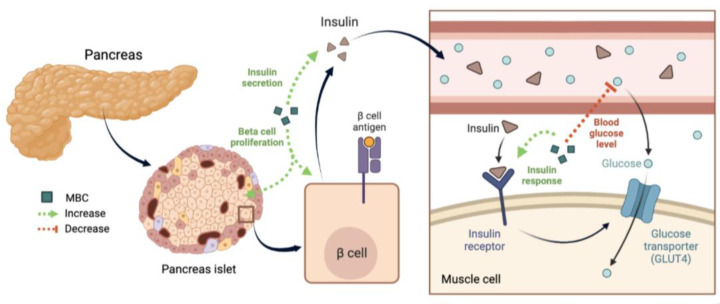
Schematic illustration of anti-diabetic properties of mushroom. Application of mushroom extracts or mushroom-derived bioactive compounds may improve insulin secretion and response by promoting pancreatic beta-cell proliferation, which increases performance of glucose take up by cells and lowering blood glucose. MBC, mushroom-derived bioactive compounds. Red dashed line indicates inhibitory effects while green dashed lines indicate promoting/strengthening effects.

Various mushroom-derived extracts and bioactive compounds, including glycoproteins and β-glucans from *Agaricus blazei* (almond mushroom), polysaccharides from *Phellinus linteus* (black hoof mushroom), lectins from *Agaricus bisporus* (white button mushroom), and extracts from *Pleurotus osteratus* (oyster mushroom) and *Ganoderma lucidum* (lingzhi), have demonstrated blood glucose reduction abilities in diabetic animal model [198,199,200,201]. Notably, lectins from white button mushrooms have been found to promote the proliferation of islet beta cells in mice with partial pancreatic removal, suggesting their potential use in the treatment of type 2 diabetes [201]. Furthermore, a retrospective study suggested that the consumption of white button mushrooms may be correlated with anti-inflammatory and anti-oxidant health benefits in individuals predisposed to type 2 diabetes [165]. Figure 4 provides an overview of the mechanisms underlying the antidiabetic activities of mushrooms.

### 4.7. Beneficial for Age-Related Diseases

Mushrooms are not only valued for their nutritional content but also considered functional foods that can enhance biological function and promote overall health [13,202,203]. Additionally, mushrooms have been found to possess pharmacological and medicinal properties that can be beneficial in age-related diseases. These properties include immunomodulatory, anti-inflammatory, anti-cancer, anti-diabetic, and neuroprotective effects, among others [13,202,204]. The following summary will highlight several representative age-related diseases or conditions that can be influenced by mushroom extracts and bioactive compounds derived from the mycelium or fruiting body of mushrooms. For a comprehensive overview of the medicinal properties of mushrooms, including the responsible compounds and proposed mechanisms, please refer to Table 5.

### 4.8. Structure–Activity Relationship

The structure–activity relationship of mushroom bioactive compounds refers to the relationship between the molecular structure of these compounds and their biological activities in addressing the mechanisms of aging. Mushroom bioactive compounds, such as polysaccharides, phenolic compounds, triterpenoids, and ergothioneine, exhibit diverse chemical structures that contribute to their bioactivity [185]. The specific structural features, such as the presence of specific functional groups or the arrangement of atoms, can influence their anti-oxidant, anti-inflammatory, and immunomodulatory properties [207,208]. Exploring the structure–activity relationship of mushroom bioactive compounds provides insights into their potential mechanisms of action and aids in the design of novel compounds with enhanced anti-aging properties.

Understanding the mechanisms and structure–activity relationship of aging is vital for developing effective interventions to mitigate the aging process and its associated diseases. Mushroom bioactive compounds have shown significant potential in addressing the mechanisms of aging through their anti-oxidant, anti-inflammatory, and immunomodulatory properties. Harnessing the power of mushroom bioactive compounds may pave the way for innovative strategies to promote healthy aging and improve the quality of life in the aging population.

## 5. Molecular and Cellular Mechanisms Underlying Aging Processes

**Figure 5:**
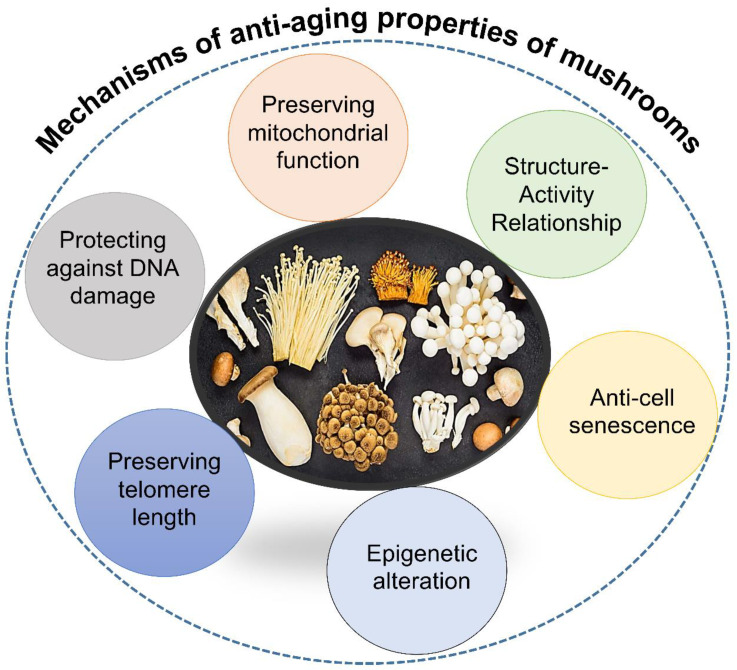
The overall mechanisms of the anti-aging properties of mushrooms.

Aging is influenced by a multitude of interconnected molecular and cellular mechanisms. These mechanisms include DNA damage and repair, telomere shortening, epigenetic changes, cellular senescence, mitochondrial dysfunction, oxidative stress, and chronic inflammation. Each of these mechanisms contributes to the aging process and the development of age-related diseases. Understanding the intricate interactions among these mechanisms is crucial for developing effective anti-aging strategies. This includes understanding how mushroom compounds interact with these mechanisms and influence their progression. The overall mechanisms of the anti-aging properties of mushrooms are depicted in Figure 5.

### 5.1. Cell Senescence

Cell senescence is a key hallmark of aging and is defined as the irreversible loss of cell division potential and the acquisition of a senescence-associated secretory phenotype. Senescent cells accumulate with age and contribute to tissue dysfunction and inflammation, which are characteristic of aging [16]. The accumulation of senescent cells has been linked to a variety of age-related diseases, including cancer, cardiovascular disease, and neurodegenerative disorders [29]. Various interventions, including the use of mushroom bioactive compounds, have been explored as potential strategies to delay or mitigate the accumulation of senescent cells and promote healthy aging [101].

Mushroom bioactive compounds have been investigated for their potential to delay or mitigate the accumulation of senescent cells and promote healthy aging. Several studies have reported the anti-senescence effects of mushroom bioactive compounds, including polysaccharides, peptides, and phenolic compounds [170,171,209]. For example, polysaccharides from *Ganoderma lucidum* (lingzhi) have been shown to reduce senescence-associated β-galactosidase activity and decrease the expression of senescence-associated markers in aging human dermal fibroblasts [171,210]. Polysaccharides from *Hericium erinaceus* (lion’s mane mushroom) have also been found to reduce senescence-associated β-galactosidase activity and increase the expression of anti-senescence markers in senescent human dermal fibroblasts [169,211].

Mushroom bioactive compounds have also been found to have anti-inflammatory and anti-oxidant properties, which may contribute to their anti-senescence effects [212]. For example, polysaccharides from *Lentinus edodes* (shiitake mushroom) have been shown to reduce oxidative stress and inflammation in aging mice, which may help delay the accumulation of senescent cells [78,213,214]. Overall, mushroom bioactive compounds have shown promise as potential interventions to delay or mitigate the accumulation of senescent cells and promote healthy aging through various mechanisms. In addition to their anti-senescence effects, mushroom bioactive compounds have been investigated for their potential to promote healthy aging through other mechanisms, such as preserving telomere length, protecting against DNA damage, and preserving mitochondrial function.

### 5.2. Telomere Maintenance

Telomeres are specific structures found at the ends of linear chromosomes. They are composed of repeated sequences of TTAGGG, known as hexanucleotides, and a protein complex called shelterin. These components work together to create a protective loop structure that prevents chromosome fusion and degradation [215]. When telomeres become shortened or damaged, and the protective loop is opened, it triggers an uncapped state that activates a DNA damage response. This response can lead to cellular senescence or programmed cell death. Traditionally, average telomere length, often measured in human blood lymphocytes, has been considered a biomarker for aging, survival, and mortality [216]. This shortening is a natural part of the aging process and is primarily caused by the inability of DNA replication machinery to fully replicate the ends of linear chromosomes. Telomerase is an enzyme that plays a critical role in maintaining telomere length, which protects the ends of chromosomes from degradation and fusion. Telomere shortening, caused by telomerase deficiency, is a hallmark of aging [217]. Shortened telomeres have been linked to cellular dysfunction, inflammation, age-related diseases and the overall decline in tissue and organ function [218].

Several factors can influence telomere maintenance and the rate of telomere shortening. These include genetic factors, lifestyle choices (such as diet, exercise, and stress levels), and environmental exposures [16,215,216,218]. Certain lifestyle modifications, such as regular physical activity, a healthy diet, and stress reduction techniques, have been associated with better telomere maintenance and potentially slower aging.

Mushroom bioactive compounds have been investigated for their potential to preserve telomere length and delay or mitigate age-related decline. While specific studies focusing on the effects of mushrooms on telomerase deficiency are limited, some studies have explored the broader anti-aging mechanisms of mushrooms that may indirectly contribute to telomere maintenance. For instance, polysaccharides from *Agaricus blazei* (almond mushroom) have been found to enhance telomerase activity and preserve telomere length in aging mice [219]. Another study found that *Ganoderma lucidum* (lingzhi) polysaccharides increased telomerase activity and extended the lifespan of fruit flies [217]. These findings suggest that mushroom bioactive compounds may have the potential to counteract telomerase deficiency and promote healthy aging.

### 5.3. Mitochondrial Dysfunction

Mitochondria are organelles responsible for producing energy in cells. Mitochondrial dysfunction, characterized by impaired energy production and increased production of ROS, is a key aspect of aging. Mitochondrial dysfunction has been linked to a variety of age-related diseases, including neurodegenerative disorders, cardiovascular disease, metabolic disorders, and impaired immune function [220,221,222]. Mitochondrial dysfunction is closely linked to the process of aging. Several factors contribute to mitochondrial dysfunction during aging. One major factor is the accumulation of mitochondrial DNA (mtDNA) mutations, which can impair the production of energy and increase the generation of harmful ROS [223]. ROS can cause oxidative damage to cellular components, including mtDNA itself, leading to a vicious cycle of further mitochondrial dysfunction [224]. It can affect various tissues and organs, including the brain, heart, muscles, and immune system.

Researchers are actively investigating strategies to mitigate mitochondrial dysfunction and its impact on aging. Approaches include improving mitochondrial quality control mechanisms, enhancing cellular anti-oxidant defenses, and exploring interventions that can promote mitochondrial biogenesis and function [225]. Understanding the complex relationship between mitochondrial dysfunction and aging is crucial for developing interventions to maintain mitochondrial health and potentially delay age-related diseases. By targeting mitochondrial function, it may be possible to enhance overall health span and improve the quality of life in older individuals.

Mushroom bioactive compounds have been investigated for their potential to preserve mitochondrial function and mitigate age-related decline. Several studies have reported the anti-mitochondrial dysfunction effects of mushroom bioactive compounds, including polysaccharides, peptides, and phenolic compounds. For example, polysaccharides from *Grifola frondosa* (maitake mushroom) have been found to preserve mitochondrial function and increase anti-oxidant enzyme activity in aging mice [226]. Polysaccharides from *Agaricus blazei* (almond mushroom) have also been shown to improve mitochondrial function and increase ATP production in aging mice [227]. The extract of *A. blazei* was found to effectively restore lipid peroxidation levels (measured by TBARS) in old rats to levels comparable to those observed in young rats [228]. This effect is likely due to the ability of various constituents in *A. blazei*, such as phenolics, to scavenge free radicals. Among the phenolics identified in *A. blazei*, gallic acid, syringic acid, and pyrogallol have demonstrated significant anti-oxidant activities [229]. Considering their hydrophilic nature, it is probable that these phenolics are present in the aqueous extract used in the study. 

In addition, the treatment with *A. blazei* was effective in elevating the activity levels of various mitochondrial enzymes in old rats, including succinate dehydrogenase, α-ketoglutarate dehydrogenase, NADH dehydrogenase, and cytochrome c oxidase. Notably, the cytochrome c oxidase activity was nearly doubled by the *A. blazei* treatment. These findings are consistent with a previous study in which old rats were treated with *Ganoderma lucidum* extracts using a similar experimental protocol [230]. In addition, the *A. blazei* treatment resulted in improved membrane energization of the mitochondrial membrane, both in the presence of succinate and ATP [231]. Succinate-driven respiration in the presence of exogenous ADP was significantly increased, approaching the respiration rates observed in the brain mitochondria of young rats. This effect is likely due to the stimulation of succinate dehydrogenase by the *A. blazei* treatment, which represents a benefit in terms of rat brain energetics. The aqueous extract of *A. blazei* has shown potential in improving the oxidative state of brain tissue and reversing certain detrimental effects of aging on mitochondrial oxidative enzymes [231]. Overall, mushroom bioactive compounds have shown promise as potential interventions to preserve mitochondrial function and mitigate age-related decline through various mechanisms.

### 5.4. DNA Damage

DNA damage is a natural consequence of aging. Over time, the genetic material in our cells can accumulate various types of damage, such as DNA strand breaks, oxidative damage, and the formation of DNA adducts. This damage can result from both endogenous factors, such as metabolic processes and ROS, as well as exogenous factors, such as exposure to environmental toxins and radiation [221,232,233]. The accumulation of DNA damage is believed to contribute to the aging process and age-related diseases. When DNA damage is not properly repaired, it can lead to mutations and genomic instability, which can affect cellular function and increase the risk of diseases such as cancer. Various mechanisms are in place to repair DNA damage, such as base excision repair, nucleotide excision repair, and homologous recombination. However, as we age, the efficiency of these repair mechanisms can decline, leading to a higher accumulation of unrepaired DNA damage [234,235,236]. Additionally, chronic inflammation and oxidative stress, which are associated with aging, can further contribute to DNA damage. These processes can generate ROS that can directly damage DNA and interfere with DNA repair mechanisms. DNA damage accumulates with age and contributes to cellular dysfunction. DNA damage can be caused by a variety of factors, including oxidative stress, radiation, and environmental toxins. DNA damage has been linked to a variety of age-related diseases, including cancer, cardiovascular disease, and neurodegenerative disorders [237,238,239].

Mushroom bioactive compounds have been investigated as potential interventions to protect against DNA damage and promote healthy aging. Several studies have reported the anti-DNA damage effects of mushroom bioactive compounds, including polysaccharides, peptides, and phenolic compounds [238]. Polysaccharides from *Phellinus linteus* (black hoof mushroom) have also been shown to protect against DNA damage in aging mice [169]. Furthermore, polysaccharides from *Grifola frondosa* (maitake mushroom) have been found to protect against DNA damage and increase anti-oxidant enzyme activity in aging mice [192]. Polysaccharides from *Ganoderma lucidum* (lingzhi) have also been shown to protect against DNA damage in human liver cells exposed to oxidative stress [240]. Mushroom bioactive compounds have also been found to have anti-oxidant and anti-inflammatory properties, which may contribute to their anti-DNA damage effects [241]. For example, polysaccharides from *Pleurotus ostreatus* (oyster mushroom) have been shown to reduce oxidative stress and inflammation in aging mice, which may help protect against DNA damage [242]. As mentioned earlier in Section 4.3, gut microbial fermentation product of *P. eryngii* (king oyster mushroom), which carries higher bioactivity than pre-fermentated original substrate, also exerts genoprotective effect via the metabolites-gut microbiome-host pathway, illustrated by its ability to protect cyclophosphamide-induced DNA damage in bone marrow and whole blood cells in young and elderly female and male mice [180]. Furthermore, mushrooms contain bioactive compounds such as ergothioneine, which has been shown to have potent anti-oxidant properties that protect DNA from oxidative damage [243]. Ergothioneine can be found in various mushrooms, including oyster mushrooms, shiitake mushrooms, and king trumpet mushrooms. Overall, mushroom bioactive compounds have shown promise as potential interventions to protect against DNA damage and promote healthy aging through various mechanisms. 

### 5.5. Epigenetic Changes

Epigenetic changes refer to modifications in gene expression that do not involve alterations to the underlying DNA sequence. These changes can have a significant impact on aging and age-related diseases. One of the key epigenetic changes associated with aging is DNA methylation. DNA methylation involves the addition of a methyl group to the DNA molecule, typically at specific sites called CpG sites [244]. Methylation patterns can change over time, and certain regions of the genome can become more methylated or less methylated with age. Global DNA hypomethylation, which is a decrease in overall DNA methylation levels, is commonly observed in aging tissues. This hypomethylation can lead to genomic instability and the activation of normally silenced genes. On the other hand, specific genomic regions, such as gene promoters, can become hypermethylated with age, resulting in the repression of gene expression [245].

Another important epigenetic modification associated with aging is histone modification. Histones are proteins that help package DNA into a compact structure called chromatin. Different modifications, such as acetylation, methylation, and phosphorylation, can occur on histones and influence gene expression [246]. Age-related changes in histone modifications can impact gene expression patterns and cellular function. For example, decreased histone acetylation levels have been observed in aging tissues, leading to a more compact chromatin structure and reduced gene expression. These epigenetic changes can be influenced by various factors, including environmental factors, lifestyle choices, and genetic predisposition [247]. They can have wide-ranging effects on cellular processes, such as DNA repair, cellular senescence, and inflammation, which are all associated with aging and age-related diseases [248].

While specific studies on the effects of mushrooms on epigenetic changes and aging are limited, some research suggests that mushroom bioactive compounds may have potential anti-aging effects through epigenetic mechanisms. For example, a study demonstrated that polysaccharides from *Ganoderma lucidum* (lingzhi) can inhibit DNA methyltransferase activity, leading to DNA hypomethylation and reactivation of tumor suppressor genes in cancer cells [249]. This suggests that mushroom polysaccharides may influence epigenetic processes that regulate gene expression. Additionally, certain mushroom bioactive compounds have been found to modulate histone modifications. For instance, extracts from *Trametes versicolor* (Turkey tail mushroom) have been shown to increase the acetylation of histone proteins, which can result in changes in gene expression [250]. These changes in histone modifications may have implications for aging and age-related diseases. Furthermore, some mushrooms contain microRNAs, which are small non-coding RNA molecules that can regulate gene expression. For instance, *Pleurotus ostreatus* (oyster mushroom) has been found to contain microRNAs that have anti-inflammatory effects by targeting specific genes involved in inflammation [251]. As chronic inflammation is associated with aging, the anti-inflammatory effects of mushroom microRNAs may have potential anti-aging benefits. It is important to note that the field of epigenetics and the effects of mushrooms on epigenetic changes and aging are still emerging areas of research.

### 5.6. Chronic Low-Grade Inflammation

Inflammation and aging are interconnected processes that have been the subject of extensive research in recent years. Chronic low-grade inflammation, often referred to as “inflammaging”, is now recognized as a hallmark of aging. As we age, our immune system undergoes changes, leading to a state of chronic inflammation. This persistent low-level inflammation can contribute to the development of various age-related diseases, including cardiovascular disease, neurodegenerative disorders, and certain types of cancer [252].

Several factors contribute to the age-related increase in inflammation. One of the key factors is the accumulation of senescent cells in tissues throughout the body. Senescent cells are damaged or dysfunctional cells that no longer divide and can produce pro-inflammatory molecules. Their accumulation over time contributes to chronic inflammation [253]. Another factor is the dysregulation of the immune system with age. This dysregulation, often referred to as immunosenescence, leads to a state of chronic immune activation and increased production of pro-inflammatory cytokines [254].

Additionally, changes in the gut microbiota, the collection of microorganisms residing in our intestines, have been linked to age-related inflammation. Alterations in the composition of the gut microbiota can lead to increased gut permeability and the release of bacterial components into the bloodstream, triggering an immune response and inflammation [255]. The consequences of chronic inflammation in aging are far-reaching. In addition to contributing to the development of age-related diseases, inflammation can also accelerate the aging process itself. It can lead to tissue damage and impair the function of organs, such as the brain, heart, and joints [256].

Efforts to mitigate age-related inflammation are being actively explored. Lifestyle factors, such as regular exercise, a healthy diet, and stress management, have been shown to reduce inflammation and promote healthy aging [257,258,259]. Certain medications and dietary supplements, such as anti-inflammatory drugs and anti-oxidants, are also being studied for their potential to modulate age-related inflammation [260]. Understanding the complex relationship between inflammation and aging is crucial for developing interventions that can promote healthy aging and reduce the burden of age-related diseases. Ongoing research in this field holds promise for improving the quality of life in older adults.

Mushrooms, particularly certain species, have been investigated for their potential anti-inflammatory properties and their ability to mitigate inflammaging. Here is some comprehensive information on inflammation and the anti-aging mechanisms of mushrooms: Mushrooms contain bioactive compounds, including polysaccharides, phenolic compounds, and triterpenoids, that have demonstrated anti-inflammatory effects [261]. For example, polysaccharides from various mushroom species, such as *Ganoderma lucidum* (lingzhi), *Lentinula edodes* (shiitake), and *Pleurotus ostreatus* (oyster mushroom), have been shown to inhibit pro-inflammatory cytokines, such as tumor necrosis factor-α (TNF-α) and interleukin-6 (IL-6) [261,262,263]. These compounds can help reduce inflammation and associated tissue damage. 

Furthermore, mushrooms have been found to modulate the immune response, which is closely linked to inflammation. For instance, mushroom polysaccharides have been shown to enhance the activity of natural killer cells, macrophages, and other immune cells, thus promoting a balanced immune response, and reducing chronic inflammation [264]. Oxidative stress plays a significant role in inflammation and aging. Mushrooms contain various anti-oxidants, including phenolic compounds and ergothioneine, which can scavenge free radicals and reduce oxidative damage. Ergothioneine, specifically found in mushrooms like *Pleurotus eryngii* (king trumpet mushroom) and *Lactarius deliciosus* (saffron milk cap), has been shown to possess potent anti-oxidant and anti-inflammatory properties [265,266].

Mushroom bioactive compounds can modulate signaling pathways involved in inflammation. For instance, polysaccharides from mushrooms like *Grifola frondosa* (maitake) and *Agaricus bisporus* (white button mushroom) have been found to inhibit the nuclear factor-kappa B (NF-κB) pathway, which is a key regulator of inflammation [267,268]. By suppressing NF-κB activation, mushrooms may help alleviate chronic inflammation. It is worth noting that while mushrooms have shown promising anti-inflammatory effects, more research is needed to fully understand their mechanisms and establish their efficacy and safety in the context of aging and age-related diseases.

## 6. Concluding Remarks and Future Perspective

As the population ages, there is an increasing demand for strategies to promote healthy aging. Dietary interventions and nutrient supplementation have been identified as effective ways to extend both health span and lifespan among the elderly. Among various food sources, mushrooms have demonstrated promising anti-aging potential due to the presence of bioactive compounds such as polysaccharides, proteins and peptides, lipids, and phenolic compounds, which have been shown to have anti-inflammatory, anti-oxidant, immunomodulatory, neuroprotective, anti-diabetic, and cardiovascular disease-ameliorating properties. 

Mushrooms can be used as functional foods and may serve as valuable source materials for drug and functional food development. While the majority of studies have used mushroom extracts in aging models and demonstrated their effectiveness in expanding lifespan, a minority of studies have identified individual compounds responsible for their anti-aging properties. It is important to identify the chemical structure of these compounds to gain insight into how they interact with cells and develop more effective anti-aging strategies. However, most studies have been performed in vivo or in vitro, with limited clinical trials, and results from different studies are not always consistent or supportive. Furthermore, individuals who consume mushrooms may also consume a variety of different self-selected meals or prepare mushrooms in different ways, which may counteract the proposed health benefits of mushroom bioactive compounds and limit their effectiveness. Therefore, meal plans for healthy aging should be designed with this factor in mind. Additionally, safety, dosage, and effectiveness of the bioactive compounds should be verified. If mushroom extracts are to be applied in the treatment of diseases among the elderly, particularly vulnerable populations, further research, particularly clinical or nutritional trials, will be highly required.

## Figures and Tables

**Table 1:** Bioactive carbohydrates in selected mushrooms.

Mushrooms	Common Names	Bioactive Compounds	Source and Yield	Bioactivities	References
*Agaricus bisporus*	Button mushroom	Heteropolysaccharide Abnp1001, Abnp1002, Abap1001, Abap1002	Concentrated industrial wastewater of *A. bisporus*; 0.989 mg/g, 1.849 mg/g, 0.128 mg/g, and 0.68 mg/g (Abnp1001, Abnp1002, Abap1001, Abap1002)	Hepatoprotective	[65]
		Heteropolysaccharide AcAPS, AcAPS-1, AcAPS-2, AcAPS-3, with rhamnose and glucose as major monosaccharide	Dried fruiting body; yield n.s.	Hepatoprotective, nephroprotective, antioxidative	[66]
		Polysaccharide extracts, main components n.s.	Whole mushroom; yield n.s.	Anti-tumor, immunostimulatory	[67]
		Heteropolysaccharide/Mannogalacoglucan mannose, galactose, glucose	Freeze-dried fresh fruiting body; 41.4% yield (*w*/*w* dry weight)	Anti-tumor	[68]
		β-glucan	Dried fresh fruiting body; yield n.s.	Immunostimulatory	[69]
		Fructose, mannitol, trehalose	Fresh fruiting body; 5.79% (white mushroom) & 4.27% (brown mushroom) (*w*/*w* fresh weight)	n.s.	[70]
*Calocybe* *indica*	Milky mushroom	Polysaccharide extracts, main components n.s.	Fresh fruiting body; 3.27% (*w*/*w* dry weight)	Anti-oxidant, neuroprotective	[71]
*Flammulina* *velutipes*	Enoki/Golden needle mushroom	Polysaccharide extracts, main components n.s.	Base of stipe; yield n.s.	Anti-tumor	[72]
		Polysaccharide extracts, main components n.s.	Fresh whole-mushroom; yield n.s.	Neuroprotective	[73]
		Fructose, mannitol, sucrose, trehalose	Fresh fruiting body; 8.29% (*w*/*w* fresh weight)	n.s.	[70]
*Ganoderma* *lucidum*	Ling Zhi	Polysaccharide extracts, main components n.s.	Mycelia; 71.99% (*w*/*w* dry weight)	Anti-inflammation, ameliorating insulin resistance, suppressing lipid accumulation, regulation of gut microbiota	[74]
		Polysaccharide extracts, main components n.s.	Commercialized spray dried mycelia; 91.48% (*w*/*w* dry weight)	Improving intestinal barrier functions	[75]
		Arabinose, galactose, glucose, xylose	Whole mushroom; yield n.s.	Anti-tumor	[76]
		Polysaccharide extracts, main components n.s.	Dried conidial powder; 2% (*w*/*w* dry weight, crude extracts)	Promote cognitive function and neural progenitor proliferation	[77]
*Lentinula edodes*	Shiitake mushroom	Glucose, galactose, mannose, arabinose	Fruiting body; 1.3% (*w*/*w* dry weight, purified polysaccharide cLEP1)	Therapeutic to cervical carcinoma	[78]
		Rhamnose	Residue/byproduct; yield n.s.	Anti-inflammatory, anti-oxidant	[79]
		Pyranose, β-d-glucans (β-(1→3)-D-glucose as backbone & β-(1→6)-D-glucose as side chains)	Dried fruiting body; 0.76% (*w*/*w* dry weight)	Anti-tumor	[80]
		Mannogalactoglucan-type polysaccharides WPLE-N-2, WPLE-A0.5-2	Fruiting body; yield n.s.	Anti-cancer, immunomodulatory	[81]
		Lentinan (β-(1,3)-glucan with β-(1,6) branches)	Dried fruiting body (commercial product); 2.6% (*w*/*w* dry weight)	Anti-tumor	[82]
		Mannitol, trehalose, arabinose	Dried powder; 23.3% (mannitol), 13.2% (trehalose), 1.79% (arabinose) (*w*/*w* dry weight)	n.s.	[83]
*Pleurotus* *eryngii*	King oyster mushroom	Mannose, glucose, galactose	Fresh whole-mushroom; 5.4% (*w*/*w* dry weight)	Anti-tumor	[84]
		Heteropolysaccharides, novel fractions PEPE-1, PEPE-2, PEPE-3 (mannose, glucose, galactose, xylose)	Fresh mushroom residue; yield n.s.	Anti-tumor	[85]
		Mannose, glucose, galactose	Fresh whole-mushroom; 28.3% (*w*/*w* dry weight)	Immunomodulatory	[86]
*Pleurotus* *ostreatus*	Oyster mushroom	Crude polysaccharide extracts	Fresh whole-mushroom; 61% (*w*/*w*)	Alleviation of cognitive impairment	[87]
		Crude polysaccharide extracts	Fresh whole-mushroom; 63.98% (*w*/*w*)	Regulation of dislipidemia	[88]
		Homogeneous polysaccharides, fractions POMP1, POMP2, POMP3	Mycelia; yield n.s.	Anti-tumor	[89]

n.s., not specified; Abnp, *Agaricus bisporus* polysaccharides between 5 kDa and 100 kDa; Abap, *Agaricus bisporus* polysaccharides under 5 kDa; AcAPS, purified fractions of acidic-extractable polysaccharides; WPLE, mannogalactoglucan-type polysaccharides from *Lentinus edodes*; POMP, *Pleurotus ostreatus* mycelium polysaccharide.

**Table 2:** Bioactive proteins in mushrooms.

Mushrooms	Common Names	Bioactive Compounds/Substances *	Bioactivities	References
*Agaricus bisporus*	Button mushroom	Lectin	Immunomodulatory	[96]
*Cerrena unicolor*	Mossy maze polypore	Laccase	Anti-tumor	[97]
*Coprinus comatus*	Shaggy mane/chicken drumstick mushroom	Laccase	Anti-viral	[98]
*Flammulina velutipes*	Enoki/Golden needle mushroom	FIP	Anti-inflammatory	[99]
		RIP	Anti-viral	[100]
*Ganoderma applanatum*	Artist’s conk	Lectin	Anti-tumor	[101]
*Ganoderma lucidum*	Lingzhi	Laccase	Anti-viral	[102]
*Ganoderma tsugae*	Hemlock reishi	FIP	Immunomodulatory	[103]
*Hypsizygus marmoreus*	Jade mushroom	RIPs (hypsin, marmorin)	Anti-fungal, anti-tumor	[104,105]
*Inonotus baumii*	Sanghuang	Laccase	Anti-tumor	[106]
*Macrolepiota procera*	Parasol mushroom	Lectin	Anti-tumor	[107]
*Pleurotus cornucopiae*	Golden oyster	Laccase	Anti-viral, anti-tumor	[108]
*Pleurotus eryngii*	King oyster mushroom	Laccase	Anti-viral	[109]
*Pleurotus ostreatus*	Oyster mushroom	Lectin	Immunomodulatory	[110]
*Sparassis latifolia*	Cauliflower mushroom	Lectin	Anti-fungal, anti-bacteria	[111]

* Include various categories and sub-categories of proteins. FIP, fungal immunomodulatory protein. RIP, ribosome inactivating protein.

**Table 3:** Lipid profiles in mushrooms.

Mushrooms	Common Name	Total SFA (% of Total FA)	Total MUFA (% of Total FA)	Total PUFA (% of Total FA)	Measurement Techniques	References
*Agaricus blazei*	Almond mushroom	24.4	2.0	73.6	GC-FID	[83]
*Agaricus bisporus*	White button mushroom	20.3	1.4	78.3	Capillary GLC-FID	[70]
	Brown button mushroom	18.4	1.8	79.8		
*Agrocybe cylindracea*	Poplar mushroom	28.1	2.83	69.1	Capillary GLC-FID	[124]
*Boletus reticulatus*	Summer cep	21.1	40.3	38.4	GLC-FID	[118]
*Coprinus comatus*	Shaggy mane/Lawyer’s cap	23.8	11.4	64.8	Capillary GLC-FID	[124]
*Flammulina velutipes*	Enoki/Golden needle mushroom	18.5	7.2	74.3	Capillary GLC-FID	[70]
		20.7	18.6	60.7	GLC-FID	[118]
*Lactarius deliciocus*	Saffron milkcap	20.8	42.0	37.3	Capillary GLC-FID	[124]
*Lactarius salmonicolor*	Salmon milkcap	19.0	19.6	61.6	GLC-FID	[118]
*Lentinus edodes*	Shiitake mushroom	16.7	3.5	79.8	GC-FID	[83]
		15.1	2.9	82.0	Capillary GLC-FID	[70]
*Pleurotus eryngii*	King oyster mushroom	17.4	13.1	69.4	Capillary GLC-FID	[70]
*Pleurotus ostreatus*	Oyster mushroom	17.0	13.6	69.4	Capillary GLC-FID	[70]
		21.8	11.4	66.5	GLC-FID	[118]
*Polyporus squamosus*	Dryad’s saddle	25.2	34.3	40.6	GLC-FID	[118]
*Russula anthracina*	-	23.7	53.3	22.9	GLC-FID	[118]
*Laetiporus sulphureus*	Sulphur polypore	21.6	17.6	60.8	GC-FID, TLC-FID	[119]
*Suillus collinitus*	-	17.5	34.4	47.4	Capillary GLC-FID	[124]
*Tricholoma myomyces*	Grey knight mushroom	15.8	46.3	37.8	Capillary GLC-FID	[124]

SFA, saturated fatty acid. MUFA, monosaturated fatty acid. PUFA, polysaturated fatty acid. GC-FID, gas chromatography coupled with flame ionization detector. GLC-FID, gas-liquid chromatography coupled with flame ionization detection. TLC-FID, thin layer chromatography–flame ionization detection.

**Table 4:** Extractable phenolic compounds in mushrooms.

Phenolic Compound Categories	Phenolic Compounds	Mushroom Sources	References
Phenolic acids	Ferulic acid	*Agaricus brasiliensis*, *Agrocybe aegerita*, *Calocybe indica*, *Cantharellus cibarius*	[126,127,132,133,134]
	Gallic acid	*Agaricus brasiliensis*, *Agrocybe aegerita*, *Calocybe indica*, *Cantharellus cibarius*, *Ganoderma lucidum*, *Pleurotus citrinopileatus*, *Pleurotus pulmonarius*, *Russula aurora*	[126,130,132,133,134,135,136,137,138]
	Cinnamic acid	*Amanita crocea*, *Ganoderma lucidum*, *Pleurotus ostreatus*, *Suilus belinii*	[135,139,140,141]
	Caffeic acid	*Calocybe indica*, *Cantharellus cibarius*, *Hyphodontia paradoxa*, *Inonotus obliquus*, *Pleurotus citrinopileatus*, *Pleurotus pulmonarius*,	[127,130,133,134,142,143]
	*p*-Coumaric acid	*Agaricus brasiliensis*, *Agaricus subrufescens*, *Amanita crocea*, *Hyphodontia paradoxa*, *Laccaria amethystea*, *Melanoleuca cognate*, *Pleurotus ostreatus*	[56,126,129,139,140,142,144]
	*p-*Hydroxybenzoic acid	*Agaricus brasilensis*, *Amanita crocea*, *Cantharellus cibarius*, *Lactarius indigo*, *Lentinus edodes*, *Melanoleuca cognate*, *Suillus belinii*	[126,129,134,138,139,141]
	Fumaric acid	*Agaricus brasiliensis*	[126]
	Vanillic acid	*Morchella esculenta* (L.) *Pers.*, *Russula emetic*	[136,137]
	Syringic acid	*Hyphodontia paradoxa*, *Morchella esculenta* (L.) *Pers.*	[129,130,136,142]
	Protocatechuic acid	*Agrocybe aegerita*, *Calocybe indica*, *Cantharellus cibarius*, *Hyphodontia paradoxa*, *Inonotus obliquus*, *Melanoleuca*, *Morchella esculenta* (L.) *Pers.*, *Suillus belinii*, *Russula emetic*	[129,130,132,133,134,136,137,141,142]
	Rosmarinic acid	*Hyphodontia paradoxa*, *Russula aurora*, *Russula emetic*	[137,142,145]
Flavonoids	Quercetin	*Ganoderma lucidum*, *Laccaria amethystea*, *Pleurotus citrinopileatus*,	[135,143]
	Kaempferol	*Ganoderma lucidum*, *Lactarius indigo*	[135,146]
	Hesperetin	*Calocybe indica*, *Ganoderma lucidum*	[133,135]
	Naringenin	*Calocybe indica*, *Ganoderma lucidum*	[133,135]
	Catechin	*Laccaria amethystea*, *Russula emetic*	[137,144]
	Myricetin	*Cantharellus cibarius*, *Lactarius indigo*	[134,146]
	Procyanidin	*Lactarius indigo*	[146]
	Rutin	*Pleurotus citrinopileatus*, *Russula emetic*	[137,143]
Tannins	Tannic acid	*Agaricus silvaticus*, *Hydnum rufescens*, *Meripilus giganteus*, *Pleurotus citrinopileatus*, *Pleurotus ostreatus*, *Pleurotus tuber-regium*(*fries*)	[147,148,149]
Tocopherols	α-Tocopherol	*Agaricus bisporus*, *Boletus badius*, *Lepista inversa*, *Pleurotus ostreatus*, *Russula delica*	[150,151]
	β-Tocopherol	*Laccaria laccata*	[150]
	γ-Tocopherol	*Clitocybe alexandri*	[150]
	δ-Tocopherol	*Lepista inversa*	[150]

**Table 5:** Medicinal properties of mushrooms.

Properties	Mushroom Species	Bioactive Compounds	Study Type/Model/Effective Dosage	Mechanisms of Action	References
Immunomodulatory	*Agaricus blazei*	β-glucans (from pure AbM extracts or commercial mushroom extracts mixture AndoSan™ containing 85% of AbM)	Ex vivo/human whole blood/0.1–15% for 6 h; In vivo/human/20 mL thrice per day orally for 12 days	Anti-oxidant activities, enhance immune cells function and innate immune responses, trigger release of cytokines, chemokines, and leukocyte growth factors	[173]
	*Pleurotus cornucopiae*	β-glucans	Clinical trial/human/24 mg per meal for 8 weeks	Th1 phenotype potentiation via macrophage-IL-12-IFN-γ pathway, up-regulation of NK cell activity	[176]
	*Latiporus sulphureus*	Lectin (LSL4)	In vitro/RAW264.7 cells/0–650 μg·mL^−1^ (IC_50_ = 1004.6 μg·mL^−1^)	Cell phagocytosis via TLR4 signaling pathway, triggers release of NO, iNOS, TNF-α, IL-1β, IL-6, and IL-10	[177]
	*Inonotus sanghuang*	Extract containing polysaccharides and amino acids	In vivo/mice/4 and 8 mg·kg^−1^ once a day orally for 12 days	Stimulation of T lymphocytes, natural killer cells, and B cells; inhibition of cytochrome P450 isozymes	[178]
	*Ganoderma lucidum*	Polysaccharides extract (Ganoderan, heteroglycan, mannoglucan, glycopeptide)	In vivo/mice/2.5 mg·kg^−1^ intraperitoneal injection once per day for 7 days	Stimulation of TNF-α, IL-1, IFN-γ production, activate NF-κB	[205]
	*Ganoderma Microsporum*	FIP	In vitro/human alveolar epithelial A549 cells/4 and 16 μg·mL^−1^	Down-regulation of TNF-α via NF-κB pathway	[206]
Anti-cardiovascular diseases	*Tricholoma matsutake*	Functional peptides	In vivo/rats/50 mg·kg^−1^ acute oral dose	Alleviated hypertension via inhibition of angiotensin I converting enzyme	[183]
	*Pleurotus florida*	Aqueous extract containing 80% soluble fiber, 44% protein, 1.4% soluble sugars, 0.2% polyphenols (*w*/*w* dry weight)	In vivo/rats/5 and 7.5% of 100 g basal diet for 4 weeks	Suppression of hepatic biosynthesis of cholesterol by inhibiting activity of liver enzyme HMG-CoA	[186]
	*Cordyceps sinensis*	Aqueous extract containing 83.9% carbohydrates (glucose, mannose, galactose, arabinose), 11.8% protein, *w*/*w* dry weight	In vivo/mice/150 and 300 mg·kg^−1^ per day orally for 7 days	Suppression of hepatic biosynthesis of cholesterol by inhibiting activity of liver enzyme HMG-CoA	[188]
Neuroprotective	*Agaricus blazei*	Extract, composition not specified	In vivo/rats/50 mg·kg^−1^ per day intragastrically at the age of 7–23 months	Free-radical scavenging ability, cytoprotective action, antioxidation reaction	[189]
	*Hericium erinaceus*	Aqueous and ehthanol extracts, composition not specified	In vitro/HT22 mouse hippocampal neurons/ethanol extracts at 400 μg·mL^−1^	Inhibition of mitochondria-dependent apoptotic cellular signals activation; elevated CAT activity and GSH content; up-regulation of MAPK and PI3K/Akt pathway	[191]
		Extract containing erinacine A, hericenones C and D	in vivo/mice/1 mg (solubilized in water) per day for 2 months	Promoting hippocampal neurogenesis; up-regulation of lipoxin A4 and modulation of stress responsive proteins	[194]
	*Auricularia polytricha*	Ethanolic extract containing flavonoids, phenols, linoleic acid	In vitro/HT22 mouse hippocampal cells/5, 10, 20, and 40 μg·mL^−1^; In vivo/*Caenorhabditis elegans*/20, 40 μg·mL^−1^	Anti-oxidant activity via Nrf2 signaling pathway; up-regulation of *Sod1* and *Gpx* gene expressions	[190]
	*Grifola frondose*	Aqueous extract containing, β-glucan, chitin, amino acids, unsaturated fatty acids, monosaccharides	In vivo/*Saccharomyces cerevisiae*/0.2 and 0.5% in culture medium; In vivo/*Drosophila melanogaster*/0.2% in culture medium	Increase of heat shock proteins expression by inhibition of Ras/PKA pathway; reduce levels of ROS	[192]
Antidiabetic	*Tremella fuciformis*, *Phellinus baumii*	Exopolysaccharides, composition not specified	In vivo/mice/200 mg·kg^−1^ per day orally for 52 days	Improve insulin sensitivity via regulating PPAR-γ-mediated lipid metabolism	[197]
	*Agaricus bisporus*	Not specified	In vivo/rats/200 mg·kg^−1^ per day orally for 3 weeks	Stimulate secretion of insulin from pancreatic beta cells	[198]
		Lectins	In vivo/mice/10 mg·kg^−1^ for 2 weeks	Induce beta-cell proliferation	[201]
	*Phellinus linteus*	Aqueous extract containing 13.2% peptide, 82.5% carbohydrates (*w*/*w* dry weight)	In vivo/mice/30 mg·kg^−1^ intraperitoneally daily from 8 to 24 weeks of age	Inhibit expression of inflammatory cytokines (IFN-γ, IL-2, and TNF-α); up-regulation of IL-4 expression	[199]
	*Agaricus blazei* Murill	Isoflavovoids (genistein, genistin, daidzein, daidzin)	In vivo/rats/400 mg·kg^−1^ per day orally for 2 weeks	Improve beta-cell function; increase lipid peroxidation via enhanced fatty acyl CoA activity	[200]

AbM, *Agaricus blazei* Murill; IFN, interferon. NK cell, natural killer cell; LSL4, one of the lectins yields from *Latiporus sulphureus*, a glycoprotein containing 6.32% sugar; TNF, tumor necrosis factor; IL, inter-leukin; FIP, fungal immunomodulatory protein; HMG-CoA, 3-hydroxy-3-methylglutaryl coenzyme A; CAT, catalase; GSH, glutathione; MAPK, mitogen-activated protein kinase; PI3K, phosphatidylinositol 3-kinase; Akt, protein kinase B; Nrf2, nuclear factor erythroid 2-related factor 2; *Sod1*, Cu/Zn superoxide dismutase; *Gpx*, glutathione peroxidase; Ras/PKA, Ras protein/protein kinase A; PPAR-γ, peroxisome proliferator-activated receptor gamma.

## Data Availability

Not applicable.
